# Cleft Palate-Lateral Synechiae Syndrome: A Case Report

**DOI:** 10.1055/s-0044-1791766

**Published:** 2024-10-24

**Authors:** Chona Thomas, Divya K.P, Princy Raphel, Nikitha Jose

**Affiliations:** 1 Department of Plastic Surgery, Aster Royal Hospital, Ghubra, Muscat, Oman; 2 Department of Audiology & Speech Language Pathology Aster Royal Hospital, Ghubra, Muscat, Oman

**Keywords:** lateral synechiae, cleft palate-lateral synechiae syndrome, cauterization

## Abstract

Lateral synechiae (LS) are fibrous bands extending from the cleft edges to the lateral edges of the tongue. The etiology of LS is still not clear, and although they are rare, they usually coexist with cleft lip palate and other congenital anomalies. In this study, we report a case of cleft palate-lateral synechiae syndrome. In this case, the lateral synechia bands were cauterized and the patient was able to open the jaw fully. Although LS is rare, medical practitioners should have awareness regarding it so that appropriate treatment protocol can be formulated for the patients.

## Introduction

Lateral synechiae (LS) are congenital malformations wherein cord-like adhesions run from the free internal borders of the palate to the lateral parts of the tongue and the floor of the mouth, causing eating difficulties due to restricted opening of the mouth. 1 2 3 They may appear isolated but are mostly associated with other congenital anomalies. One such syndrome is known as the cleft palate-lateral synechiae syndrome (CPLSS). It was first described in 1972. 4 It is a rare syndrome that includes cleft palate, lateral synechia, and micrognathia. Some studies have noted an autosomal dominant inheritance with variable penetrance and expression. 4 In this study, we report a rare case of CPLSS wherein the patient had developed LS along with a cleft palate and micrognathia with no breathing difficulty or any other congenital anomalies. Oral synechia can be of various types according to their appearance at different locations.

## Case Report

A 7-day-old newborn female was referred from a private hospital in Muscat and was brought to our tertiary care hospital with intraoral fibrous bands along with a wide cleft palate, with micrognathia. The neonate was born through lower segment cesarean section (LSCS) to a healthy mother. The newborn is the third child of the parents and her siblings show no sign of congenital abnormalities or malformations. Also, there is no family history mentioned from both paternal and maternal sides.

### Physical Examination

**Fig. 1:**
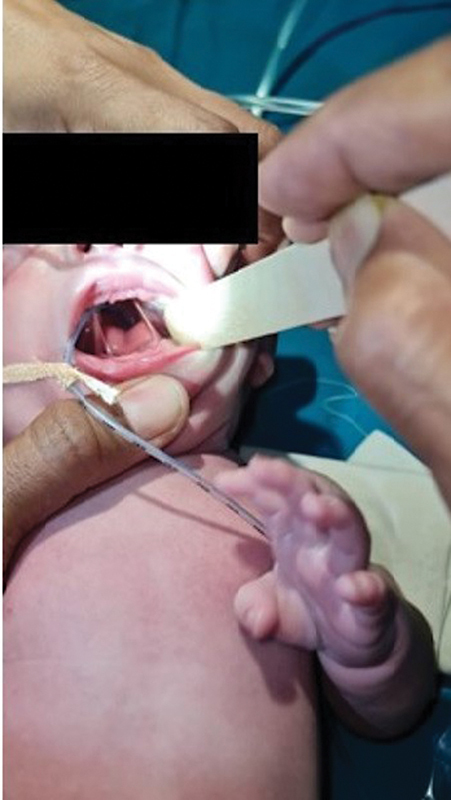
Preoperative image of an infant with lateral synechiae.

The examination of oral cavity showed multiple fibrous bands, two bands on either side attached from the lateral aspects of the tongue to the palate (LS), due to which the neonate was not able to open the mouth fully and was unable to protrude the tongue, leading to feeding difficulties. The neonate was provided with orogastric feeding tube with no breathing difficulties. Besides that, the patient also exhibited a cleft palate. Fig. 1 depicts the preoperative image of the neonate with LS.

### Intervention

**Fig. 2:**
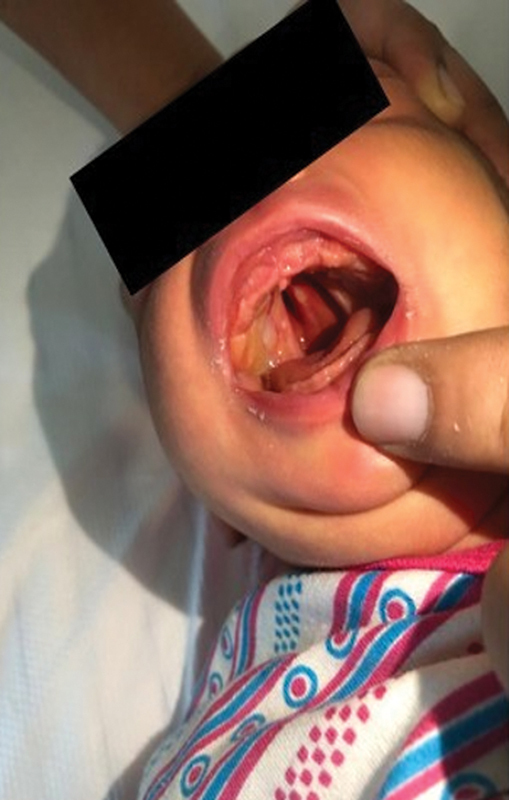
Postoperative image of an infant with lateral synechiae.

In this case, the priority was to open the patient's mouth so that the neonate could be fed properly. The patient underwent surgical excision of the bilateral fibrous bands with bipolar cauterization, to prevent bleeding, without any anesthesia in the outpatient clinic. This procedure offered a spontaneous resolution of the problem. Histopathology was not done. Fig. 2 shows the postoperative image after cauterization of the fibrous bands.

### Outcome and Further Treatment Plan

After fibrous bands were cauterized and the tongue was detached from the palate, the patient was able to fully open the jaw and was in a position to feed well. The newborn was in good condition. The parents were counseled regarding management of the cleft palate and were informed about cleft palate repair. The patient will be regularly evaluated by a multidisciplinary team for further interventions.

## Discussion

This study reported a rare occurrence of CPLSS. The presence of these lateral fibrous bands has been seldom reported in the literature. 3 5

The etiology of LS is still unknown; however, there are certain theories that explain its formation at the embryonic stage. One such theory hypothesizes that LS may have been formed from a subglossopalatal membrane that precedes the fusion of the palatal shelves, at the seventh week of embryonic development when the floor of the mouth and the palate are in close proximity. 6 7 Moreover, less than normal movement of the mandible and the tongue is assumed to prompt the formation of this subglossopalatal membrane. 6 8

There is debate about whether the continuous interposition of the tongue between the palatal shelves is a result of the intraoral synechiae causing a decrease in tongue movements or if the changes of the tongue and mandibular motilities are the outcome of the formation of intraoral synechiae due to its close contact with the buccal cavity. 6 7 8

Another hypothesis suggests that during embryogenesis, the oropharyngeal membrane separates the mouth from the pharynx and foregut. This membrane then sheds around the 26th day of intrauterine life, and the tongue lowers down around the 8 ^th^ week, which permits palatal closure at around 8 to 9 weeks of gestation. This ultimately prevents the fusion and assists in mandibular growth. Nevertheless, if the buccopharyngeal membrane does not regress and the tongue remains interposed between the palatal shelves, it may lead to an abnormal fusion, resulting in LS. 6 8

Research studies have suggested that the synechia has no impact on fetal development. However, LS is associated with feeding and swallowing difficulties in newborn as evident from the case reported in this study wherein the patient was not able to open the jaw fully, resulting in feeding difficulties. The consensus on the treatment of LS is excision of the synechia and palatal closure. Spontaneous cauterization of the fibrous bands is very effective as it helps in maintaining homeostasis, as observed in this case. Follow-up study revealed that the child is doing well and feeding normally and waiting for the cleft palate repair.

## Conclusion

This report asserts the effectiveness of surgical excision of fibrous bands with bipolar cautery in CPLSS. Medical practitioners should be aware of LS so that appropriate treatment protocol can be formulated for the patients. Future researches are warranted to understand the pathophysiology of LS, which can assist in developing appropriate management plan for such patients.
